# Awakened Beta-Cell Function Decreases the Risk of Hypoglycemia in Pregnant Women with Type 1 Diabetes Mellitus

**DOI:** 10.3390/jcm11041050

**Published:** 2022-02-17

**Authors:** Josip Delmis, Marina Ivanisevic

**Affiliations:** Clinical Department of Obstetrics and Gynecology, University Hospital Centre Zagreb, School of Medicine, University of Zagreb, 10000 Zagreb, Croatia; marina.ivanisevic@pronatal.hr

**Keywords:** C-peptide, diabetes mellitus type 1, hypoglycemia, pregnancy

## Abstract

Diabetes in pregnancy creates many problems for both the mother and child. Pregnant women with type 1 diabetes experience more frequent hypoglycemic and hyperglycemic episodes. This study aimed to determine the risk of clinically significant biochemical hypoglycemia (CSBH) by HbA1c, fasting C-peptide, mean plasma glucose (PG), and insulin dose in pregnant women type 1 diabetes mellitus according to each trimester of the pregnancy. Methods. We conducted a prospective observational study of 84 pregnant women with type 1 diabetes in an academic hospital. To present the hypoglycemia, we divided the participants into two groups: those who did not have clinically significant biochemical hypoglycemia (CSBH−; *n* = 30) and those who had clinically significant biochemical hypoglycemia (CSBH+; *n* = 54). Results. In the first, second, and third trimesters, the duration of T1DM, fasting C-peptide, and mean glucose concentration was inversely associated with CSBH. Conclusions. Insulin overdose is the most common risk factor for hypoglycemia. In pregnant women with type 1 diabetes with elevated fasting C-peptide levels, the insulin dose should be diminished to reduce the risk of hypoglycemia.

## 1. Introduction

Poor metabolic control in pregnant women with type 1 diabetes mellitus is associated with an increased risk of spontaneous abortion, preeclampsia, congenital malformations, asphyxia, macrosomia, and neonatal morbidity and mortality [1,2,3]. For a successful perinatal outcome, an intensive clinical approach is required to achieve normoglycemia before conception and pregnancy. Good metabolic control (fasting plasma glucose of 3.9–5.3 mmol/L and 1 h postprandial values between the glucose of 6.1–7.8 mmol/L or 2 h postprandial glucose of 5.6–6.7 mmol/L, and HbA1c values <6.0% (<42 mmol/mol)) exhibits potential pregnancy complications as being equal to those in the healthy pregnant population [2]. Treating women with type 1 diabetes mellitus aims to achieve normoglycemia before and during pregnancy to reduce spontaneous abortion, congenital malformations, fetal macrosomia, and neonatal complications. Tight glycemic control improves pregnancy outcomes; however, it also increases the risk of hypoglycemia [3,4] and possibly causes maternal complications, including coma, convulsion, and death [5]. Severe hypoglycemia affects up to 19–44% of pregnant women with type 1 diabetes and is 15 times higher than that observed with intensified treatment outside of pregnancy [3,6]. The risk of hypoglycemia is usually highest in early pregnancy, especially during the first trimester, due to overinsulinization [6,7].

The known risk factors for hypoglycemia are the duration of diabetes, history of previous severe hypoglycemia, hypoglycemia unawareness, change in insulin treatment, and HbA1c <6.0% (42 mmol/mol) [1,3]. A successful pregnancy outcome needs to achieve normoglycemia, with HbA1c levels between 4.0 and 6.0% (20 and 42 mmol/mol) [3,8]. Reducing the risk of hypoglycemia is a significant challenge for doctors who care for pregnant women with type 1 diabetes mellitus. The International Hypoglycemia Study Group (IHSG) considers glucose concentration levels of <3.0 mmol/L unequivocally hypoglycemic values, which are detected by self-monitoring of plasma glucose, continuous glucose monitoring (for at least 20 min), or laboratory measurement of plasma glucose [3,7]. The glycemic threshold for cognitive impairment is <2.8 mmol/L [7]. The IHSG considers a glucose concentration <3.0 mmol/L low enough to indicate severe, clinically significant hypoglycemia [8]. The same group suggested that a glucose value of 3.9 mmol/L or less should only indicate possible hypoglycemia. Severe hypoglycemia is considered a hypoglycemic episode requiring external assistance for recovery [7].

This study aimed to determine the risk of clinically significant biochemical hypoglycemia (CSBH) by HbA1c, gestational weight gain, C-peptide, mean capillary plasma glucose, and total insulin dose in pregnant women with type 1 diabetes mellitus in each trimester of pregnancy. The specific aim was to establish the effect of the C-peptide concentration on the prevalence of CSBH in pregnant women with type 1 diabetes and its association with insulin dosage.

## 2. Materials and Methods

### 2.1. Ethical Statements

The Ethics Committee of the School of Medicine, the University of Zagreb (No. 380-59-10106-19-111/26), approved the study within the scientific project PRE-HYPO No. IP-2018-01-1284. All women included in the study provided written informed consent for themselves and their newborns.

### 2.2. Study Participants

In the prospective observational study, we consecutively included 84 women with type 1 diabetes mellitus before completing 10 gestational weeks with a single living fetus during the study period from 1 January 2018 to 31 December 2019. Pregnant women without complications of diabetes and those with non-proliferative retinopathy and diabetic neuropathy were included in the study. All participants were admitted to the Department of Obstetrics and Gynecology at least once or repeatedly in each trimester. The daily glucose profiles of patients with type 1 diabetes were determined, and plasma glucose (9/day) was monitored for 2–3 days. Glucose was measured in capillary plasma at the following time intervals: 7, 10, 13, 16, 19, 22, 1, 4, and 7 h.

Clinically significant biochemical hypoglycemia was defined as a glucose concentration of ≤3.0 mmol/L detected by laboratory measurement of plasma glucose. None of the pregnant participants in this study experienced a hypoglycemic coma or needed third-party assistance during hypoglycemia or glucagon/intravenous glucose during the hypoglycemic event. No episodes of severe hypoglycemia were reported for the whole pregnancy.

We included 84 women with type 1 diabetes and singleton pregnancies who received insulin therapy for ≥2 years. At pregnancy confirmation, the HbA1c was ≤8% (≤64 mmol/mol). All pregnant women received intensified insulin therapy with fast-acting insulin aspart and long-acting insulin detemir.

We divided the participants into two groups according to the prevalence of CSBH events: into the group of participants who did not have CSBH− (*n* = 30) and into the group with CSBH+ (*n* = 54).

The maternal and umbilical vein sera were analyzed for fasting C-peptide concentration, and the HbA1c percentage, along with glucose levels, were measured in maternal blood only.

Pregnant women with T1DM who had proliferative retinopathy, nephropathy, and chronic hypertension were excluded from the study.

### 2.3. Data Collection

The following parameters were recorded: maternal height (cm) and weight (kg) before pregnancy, gestational weight gain, which was the difference in weight before pregnancy (self-reported) and at time of delivery; and the pre-pregnancy body mass index (kg/m^2^; BMI), calculated from the pre-pregnancy values.

Neonatal macrosomia was defined as ≥4000 g. Blood samples were obtained from the antecubital vein for glucose measurements when indicated, as well as HbA1c determination in each trimester throughout pregnancy in the type 1 diabetes mellitus groups. Umbilical vein blood samples were obtained immediately after birth but before the placenta was removed through puncture of the umbilical vein for glucose and C-peptide. Neonatal birth weight (g), length (cm), and the 1 min and 5 min Apgar scores were measured postnatally.

### 2.4. Blood Sample Analyses

The glucose levels were quantified by the hexokinase method on a Cobas C301 analyzer with reagents from the same manufacturer (Roche, Basel, Switzerland). The HbA1c levels in whole blood were measured by turbidimetric inhibition immunoassays on a Cobas C501 instrument (Roche, Basel, Switzerland). The C-peptide concentrations were determined by electrochemiluminescence immunoassays (ECLIA) with Elecsys immunoassay analyzers (Roche Diagnostics, Switzerland). The lower detection limit of C-peptide in serum is 0.003 nmol/L.

According to a homeostasis model assessment, neonatal insulin resistance was calculated using online software (https://homa-calculator.informer.com/2.2/, accessed on 15 January 2022).

### 2.5. Sample Size

We performed a power calculation using G*power 3.1.9.4 (https://g-power.apponic.com/, accessed on 15 January 2022). For sample size calculation, we tested the mean difference in C-peptide concentration between CSBH− and CSBH+ in the first trimester of pregnancy. For 80% power *p* < 0.05, a total sample size of 45 participants was needed.

### 2.6. Statistical Analyses

Statistical analyses were performed using the statistical package of SPSS version 24 (IBM, Armonk, NY, USA). Continuous variables are expressed as the mean ± SD, or median (25th–75th percentile) for a skewed distribution, and qualitative variables are presented as frequencies and percentages. Between-group differences in normally distributed continuous variables were assessed with Student’s *t*-test. The Mann–Whitney U test was used for variables with a skewed distribution, and the χ^2^ test was used for proportions. For repeated measurements of continuous data, the Wilcoxon signed-rank test was used. Data that were not normally distributed were log-transformed before Spearman’s nonparametric correlation analyses. Statistical tests were two-sided.

## 3. Results

### Impact of Clinically Significant Biochemical Hypoglycemia (CSBH) on Maternal and Neonatal Characteristics

The duration of diabetes is a significant risk factor for developing CSBH in pregnant women with type 1 diabetes, as demonstrated in Table 1.

The age, height, body weight, BMI, and weight gain did not differ between pregnant women and those without CSBH. We found the difference in prescribed total insulin doses between the groups with and without CSBH.

**Figure 1:**
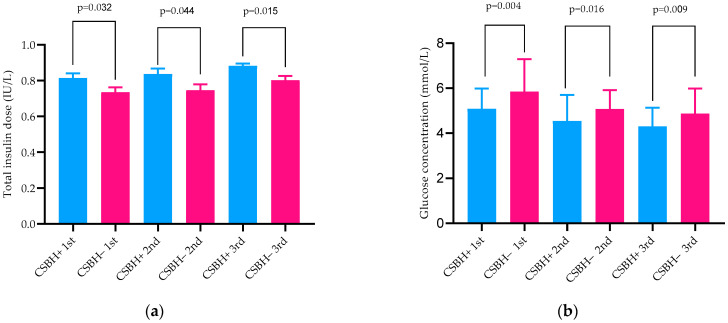
(**a**) Mean and standard deviation total insulin dose in two study groups in three trimesters of pregnancy (IU/kg). (**b**) Mean and standard deviation of glucose concentration in capillary plasma study groups in three trimesters of pregnancy (mmol/L).

In all trimesters of pregnancy, the women with CSBH had lower C-peptide levels and glucose concentration in the daily profile, as demonstrated in Table 1, Figure 1a,b. The HbA1c values were significantly lower in the second trimester in the group of pregnant women with CSBH+ (*p* = 0.004).

The newborns did not differ in gestational age, birth weight and length, Apgar index at 1 and 5 min, or macrosomia prevalence. No difference was found in the glucose, C-peptide concentration, and insulin resistance HOMA 2 between the study groups (Table 2).

A significant positive correlation was obtained between CSBH+ and the duration of T1DM (*p* < 0.05). Comparing CSBH+ with C-peptide concentration, a significant negative correlation was found in all pregnancy trimesters, as shown in Table 3. A significant negative correlation was obtained between the mean value of glucose and CSBH+: Table 3.

**Figure 2:**
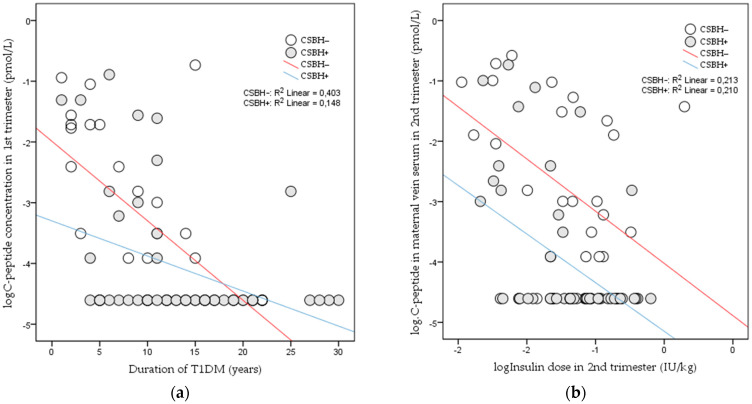
(**a**) Nonparametric linear correlation between duration of T1DM and log C-peptide concentration (CSBH+, R^2^ = −0.148, *p* = 0.005; CSBH−, R^2^ = −0.403, *p* < 0.001). (**b**) Nonparametric linear correlation between total log insulin dose and log C-peptide concentration (CSBH+: R^2^ = −0.210, *p* < 0.031; CSBH−: R^2^ = −0.213, *p* < 0.001).

The duration of T1DM is inversely correlated with C-peptide concentration: Figure 2a.

The total insulin dose is inversely correlated with C-peptide: Figure 2b.

## 4. Discussion

### 4.1. C-Peptide Concentration in Pregnant Women with Type 1 Diabetes Mellitus

The most reliable indicator of maintaining beta-cell function is the concentration of C-peptide in the blood. Measurement of the C-peptide concentration provides a validated way to quantify secreted endogenous insulin. The close association between C-peptide in the systemic circulation and endogenous insulin in the portal system is well-established [3,9,10,11]. Nielsen et al. showed that the C-peptide concentration gradually increases during pregnancy, independent of blood glucose concentration, in pregnant women suffering from type 1 diabetes mellitus [3,10]. Comparing C-peptide concentrations across all three trimesters, we found an increase in the CSBH− group from the first to the third trimester. This finding is consistent with our previous research showing that pregnancy increases the C-peptide concentration in healthy pregnant women and women with type 1 diabetes [11] and is compatible with the reported C-peptide increase throughout pregnancy (Nielsen et al.) [3,10]. As the C-peptide does not cross the placenta in either direction, the high C-peptide values detected during pregnancy originate from the maternal beta-cells rather than the fetus.

The increase in the C-peptide concentration in both groups of pregnant women might be mirrored by suppression of the inflammatory immune system during pregnancy, which enhances the ability of the mother to have a genetically and immunologically diverse fetus [3,12,13]. Consequently, the mother’s immune system undergoes significant changes, including developing several specific pathways to protect the fetus from maternal cytotoxic attack. One mechanism reduces the expression of classical HLA class I molecules, while the other mechanisms are associated with an altered Th1 and Th2 balance [3,12]. Cellular immune function and pro-inflammatory Th1 cytokines (e.g., IL-2, TNF-α, and INF-γ) are suppressed during pregnancy. In contrast, humoral immunity and the production of anti-inflammatory Th2 cytokines (e.g., IL-4 and IL 10) are enhanced. This immune function pattern is reversed in the postpartum period [12,13]. A partial decrease in the activity of the inflammatory immune system leads to the suppression of various autoimmune diseases during pregnancy, including diabetes. In type 1 diabetes, these changes are expressed through the growth and functional modification of the Langerhans pancreatic islets. The most significant difference that the Langerhans islets undergo during pregnancy is insulin secretion enhancement or improved beta-cell proliferation [3,14].

Numerous animal model studies have shown that the beta-cell mass increases 3–4 times during pregnancy. In addition to significant maternal beta-cell hypertrophy, beta-cell proliferation during pregnancy dramatically increases [14,15]. Nielsen et al. [10] observed a rapid decrease in the postpartum C-peptide concentration. This finding also indicates the active role of the placenta in increasing the concentration of C-peptide during pregnancy. Placental growth factors and hormones that reduce maternal lymphocyte response of the fetoplacental unit [15,16] and beta-cell hyperplasia are no longer excreted after pregnancy.

The data acquired in this study are consistent with earlier studies because the concentration of C-peptide in both study groups was higher in the third trimester than in the first trimester. Tight glycemic control during pregnancy was mirrored by a significant decline in the HbA1c percentage in the third trimester, with average values below 6%. The strict glycemic control also resulted in a low rate of macrosomic infants, although the high percentage of severe hypoglycemia continued during pregnancy.

### 4.2. C-Peptide, Insulin Doses, and Glycemic Control

We compared the insulin dose between the first and third trimesters. We found that they were significantly reduced with a simultaneous increase in the C-peptide levels in both groups [3] (Figure 2b).

Earlier studies have shown that improved glycemic control during pregnancy leads to increased C-peptide concentrations in pregnant women with type 1 diabetes [16,17]. Although they found no association between the reduced insulin doses at the end of pregnancy and the increased C-peptide concentrations, Nielsen et al. concluded that improved glycemic control facilitates C-peptide production [10]. The authors believe that achieving and maintaining reasonable metabolic control in pregnancy plays a role in beta-cell regeneration. However, our study showed that the C-peptide concentrations affect the insulin dose. A higher C-peptide concentration decreases the total insulin dose, and a lower C-peptide level increases the total insulin dose. We obtained these results thanks to accurate data on the need for daily insulin doses and the determination of glucose and C-peptide concentrations in the same hospital laboratory. So far, only Ilic et al. [18] found a decrease in insulin dose with increased C-peptide concentration in the first trimester of pregnancy.

However, the results of this study did not show a significant correlation between the HbA1c percentage and fasting glucose with the serum C-peptide concentrations. Although this result may seem unexpected, the reason may be that pregnant women in both groups had well-controlled glycemia.

Preservation of beta-cell function decreases the number of hypoglycemic events during pregnancy. It improves metabolic control and reduces the risk of long-term diabetic complications and the adverse effects of intensive therapy, primarily hypoglycemia [3,18,19].

The umbilical vein glucose and C-peptide concentrations differed between the study groups. In a previous study, Delmis et al. showed a significant positive association between the glucose and insulin levels in the umbilical vein [20], that is, increased glucose concentrations increased the synthesis and release of fetal insulin and vice versa. In this study, the concentration of C-peptide in the umbilical vein was correlated with the umbilical vein glucose level.

This research has its advantages and limitations. To the best of our knowledge, this is the first study to investigate the effect of C-peptide on the prevalence of severe hypoglycemia and the insulin dose in pregnant women with type 1 diabetes mellitus. A strength of this report is that this is a prospective study with evidence of fasting glucose and C-peptide values in capillary plasma and 8-point glucose profiles determined in the same hospital laboratory with accurate fast-acting and long-acting insulin dose data. Another advantage is the determination of glucose in the maternal capillary plasma and the glucose and C peptides in the umbilical vein immediately after delivery while the placenta was still in situ. The potential limitation of this study is that no C-peptide values were determined postprandially.

## 5. Conclusions

Although insulin overdose is the most common risk factor for hypoglycemia, there are more subtle endogenous actors in diabetic pregnancy, which impact hypoglycemia occurrence. An increase in fasting C-peptide in the CSBH group occurred in the third trimester of pregnancy compared to the first. The lower prevalence of clinically significant biochemical hypoglycemia and the decrease in the required dose of insulin during pregnancy were associated with increased endogenous insulin secretion. The duration of pregnancy serves as a mediator between CSBH and C-peptide. Our suggestion would be to introduce fasting and postprandial C peptide as additional valuable laboratory parameters in the conventional clinical approach as a complementary tool for insulin dose titration throughout diabetic pregnancy.

## Figures and Tables

**Table 1:** Maternal characteristics according to groups and trimesters of pregnancy.

	CSBH− (*n* = 30)	CSBH+ (*n* = 54)	*p*
Maternal characteristics in 1st trimester			
Maternal age (years)	29.8 ± 5.3	29.3 ± 6.3	0.693
Maternal height (cm)	166.3 ± 7.4	166.7 ± 6.9	0.798
Maternal pre-pregnancy weight (kg)	63.5 ± 10.9	63.8 ± 8.0	0.804
Pre-pregnancy body mass index (kg/m^2^)	22.8 ± 2.9	22.9 ± 2.8	0.755
Gestational weight gain (kg)	13.3 ± 4.4	13.5 ± 4.8	0.858
Duration of type 1 diabetes mellitus (years)	10.1 ± 6.6	13.5 ± 7.1	0.032
Total insulin dose (IU/kg) 1st trimester	0.74 ± 0.15	0.82 ± 0.18	0.044
Total insulin dose (IU/kg) 2nd trimester	0.75 ± 0.18	0.84 ± 0.21	0.045
Total insulin dose (IU/kg) 3rd trimester	0.80 ± 0.13	0.89 ± 0.17	0.015
Maternal vein blood (serum and plasma) measurements in 1st trimester of pregnancy			
HbA1c % (mmol/mol)	6.8 ± 1.3 ** (51)	6.7 ± 1.0 ** (50)	0.296
Fasting C-peptide (pmol/L)	180.0 * (90.0–230.0)	50.0 (30.0–70.0)	0.005
Fasting glucose (mmol/L)	5.1 ± 1.9	5.0 ± 2.0	0.870
Mean glucose concentration (mmol/L)	5.9 ± 1.4	5.1 ± 0.9	0.004
Maternal vein blood (serum and plasma) measurements in 2nd trimester of pregnancy			
HbA1c % (mmol/mol)	5.9 ± 0.5 (41)	5.5 ± 0.6 (37)	0.004
Fasting C-peptide (pmol/L)	130 (80–220)	60 (30–90)	0.004
Fasting glucose (mmol/L)	5.0 ± 1.8	4.4 ± 1.8	0.165
Mean glucose concentration (mmol/L)	5.9 ± 1.4	5.1 ± 0.9	0.016
Maternal vein blood (serum and plasma) measurements in 3rd trimester of pregnancy			
HbA1c % (mmol/mol)	6.9 ± 0.7 ** (52)	5.8 ± 0.8 ** (40)	0.142
Fasting C-peptide (pmol/L)	210 (130–240) *	60 (30–90)	0.001
Fasting glucose (mmol/L)	5.1 ± 1.4	4.8 ±1.8	0.508
Mean glucose concentration (mmol/L)	5.3 ± 1.0	4.6 ± 1.3	0.009

CSBH—Clinically Significant Biochemical Hypoglycemia; Wilcoxon test * *p* < 0.05; ** *p* < 0.001.

**Table 2:** Neonatal characteristics.

	CSBH− (*n* = 30)	CSBH+ (*n* = 54)	*p*
Gestational age at delivery (weeks)	38.5 ± 0.7	38.3 ± 1.1	0.488
Birth weight (g)	3573.0 ± 542.1	3449.3 ± 421.2	0.231
Birth length (cm)	49.6 ± 2.1	49.0 ± 1.8	0.178
Ponderal index	2.9 ± 0.2	2.9 ± 0.3	0.822
Fetal macrosomia >4000 g n Yes/No (%)	7/23 (23.3/76.7)	5/49 (9.3/90.7)	0.071
Apgar score at 1 min	9.7 ± 1.0	9.9 ± 0.4	0.217
Apgar score at 5 min	9.8 ± 0.5	9.9 ± 0.2	0.157
Umbilical vein serum measurements			
C-peptide (pmol/L)	580.0 (340.0–1100.0)	850.0 (580.0–1250.0)	0.056
Umbilical vein glucose mmo/L	4.7 ± 1.5	4.6 ± 1.4	0.428
IR HOMA 2	1.9 (0.9–2.8)	2.1 (1.4–2.9)	0.492

**Table 3:** Nonparametric correlations between the CSBH duration of T1DM, C-peptide and mean glucose concentration according to the trimesters of pregnancy.

	CSBH+	Duration of T1DM
DurationT1DM	0.224 *	
Mean glucose concentration in 1st trimester of pregnancy	−0.375 **	−0.073
Mean glucose concentration in 2nd trimester of pregnancy	−0.256 *	−0.028
Mean glucose concentration in 3rd trimester of pregnancy	−0.387 **	−0.115
C-peptide in 1st trimester of pregnancy	−0.331 **	−0.552 **
C-peptide in 2nd trimester of pregnancy	−0.332 **	−0.564 **
C-peptide in 3rd trimester of pregnancy	−0.314 **	−0.546 **

* *p* < 0.05; ** *p* < 0.01.

## Data Availability

Data can be found on URL https://figshare.com/s/971031f799498beb2274, accessed on 15 January 2022.
